# Update in short-term mechanical circulatory support: indications, devices and complications

**DOI:** 10.3389/fcvm.2026.1994496

**Published:** 2026-10-22

**Authors:** Jose María Moreno-Coca, Eva Moreno-Monterde, Antoni Manzano, Rut Andrea, Marc Giménez-Milà

**Affiliations:** 1 Acute Cardiac Care Section, Department of Cardiology, Institut Clínic Cardiovascular, Hospital Clínic de Barcelona, Universitat de Barcelona, Barcelona, Spain; 2 Institut D'Investigacions Biomèdiques August Pi I Sunyer (IDIBAPS), Barcelona, Spain; 3 Department of Anaesthesiology and Intensive Care, Hospital Clínic de Barcelona, Barcelona, Spain

**Keywords:** cardiogenic shock, IABP, intensive cardiovascular care, mechanical circulatory support, microaxial flow pump, VA-ECMO

## Abstract

Despite advances in early recognition, aetiology-directed therapy, intensive cardiovascular care and short-term mechanical circulatory support (MCS), cardiogenic shock (CS) remains associated with high mortality and morbidity. MCS can restore systemic perfusion and stabilise patients as a bridge to recovery or advanced heart failure therapies, or as a bridge-to-decision when the final therapeutic destination remains uncertain. However, appropriate patient and device selection remains challenging. The optimal timing of support is uncertain, as both premature implantation and delayed use in advanced shock may limit benefit while increasing complications. Device selection should therefore be individualised according to the predominant patient phenotype, including left ventricular, right ventricular or biventricular failure, concomitant respiratory failure, vascular access and anatomical considerations. Randomised trials have focused predominantly on acute myocardial infarction-related CS, whereas data in heart failure-related and acute-on-chronic CS remain limited. Recent trials have also shown that greater haemodynamic support does not uniformly translate into improved survival, highlighting the importance of matching device physiology to shock phenotype and clinical trajectory. MCS is associated with clinically relevant complications, including major bleeding, haemolysis, limb ischaemia, stroke, thrombosis and vascular injury. Accordingly, its use should be considered within a strategy integrating reversibility, neurological prognosis, recovery potential, a realistic definitive therapeutic strategy, technical feasibility and anticipated complication risk. This Mini Review summarises contemporary indications, devices and complications of MCS in CS, with emphasis on phenotype-guided selection, timing, intensive care management and current evidence gaps.

## 1 Introduction

Cardiogenic shock (CS) remains associated with high short-term mortality despite advances in revascularisation, critical care, pharmacological therapy and short-term mechanical circulatory support (MCS) (1, 2). It is increasingly recognised as a heterogeneous syndrome encompassing different aetiologies, severities, clinical trajectories and patterns of ventricular involvement (1–3).

This heterogeneity has major therapeutic implications, as patients with acute myocardial infarction-related CS (AMI-CS), *de novo* heart failure-related CS (HF-CS) and acute-on-chronic HF-CS may differ in haemodynamic profile, recovery potential and suitability for advanced heart replacement therapies (3, 4). Epidemiological patterns vary across cohorts and healthcare systems. HF-CS represented 69% of ventricular-failure CS in a contemporary North American cardiac intensive care registry, whereas AMI-CS accounted for 60.7% of all CS cases in the Catalonian Shock-CAT cohort, reflecting differences in study populations and case mix (5, 6).

MCS can augment systemic perfusion, unload the failing ventricle or provide combined circulatory and respiratory support. It may serve as a bridge to myocardial recovery or advanced heart failure therapies, or as a bridge-to-decision when the final therapeutic destination remains uncertain (4, 7). However, improved haemodynamics do not necessarily translate into better outcomes, and device-related complications may offset benefit (3, 4, 7, 8).

The key questions are who is most likely to obtain clinical benefit, when support should be initiated, and which device best matches the underlying pathophysiology.

These decisions require integration of shock severity and trajectory, ventricular and respiratory phenotype, vascular and anatomical feasibility, comorbidity and frailty, recovery potential and a realistic therapeutic strategy (2, 4, 7).

Evidence remains particularly limited in non-ischaemic and HF-related CS (4, 9). This Mini Review complements recent consensus documents by integrating dynamic shock phenotype, clinical trajectory, therapeutic destination and complication risk into a practical bedside framework for MCS selection, reassessment and de-escalation, while distinguishing physiological rationale from the strength of available clinical evidence.

## 2 Cardiogenic shock phenotypes and indications for MCS

Cardiogenic shock is a dynamic syndrome in which tissue hypoperfusion cannot be inferred from arterial pressure alone. Normotensive CS may occur when increased systemic vascular resistance (SVR) preserves systolic blood pressure >90 mmHg despite hypoperfusion, while inflammation, myocardial necrosis or cardiac arrest may subsequently promote vasoplegia (1). Mixed cardiogenic–vasodilatory shock is a haemodynamic phenotype characterised by cardiac dysfunction with inadequate forward flow, systemic hypoperfusion and an inappropriate vasodilatory component, often reflected by reduced SVR (10). Recognition is clinically relevant because restoring cardiac output alone may not adequately correct systemic hypoperfusion (Figure 1).

**Figure 1:**
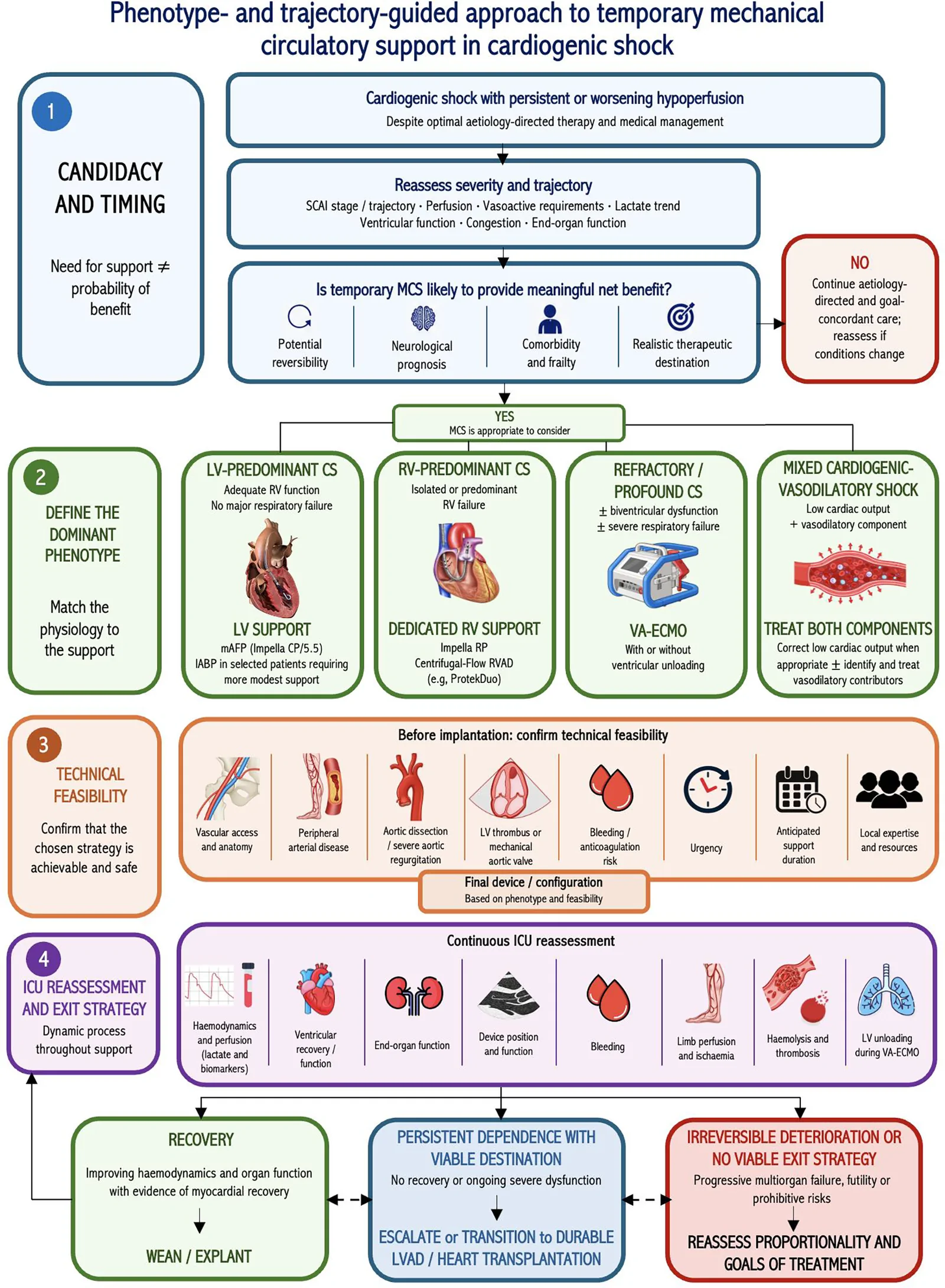
Phenotype- and trajectory-guided approach to MCS in cardiogenic shock. MCS should be considered within a dynamic strategy integrating shock severity and trajectory, reversibility, neurological prognosis and a realistic therapeutic destination. When support is considered appropriate, device selection should address the predominant ventricular and respiratory phenotype while accounting for vascular and anatomical feasibility. Clinical, haemodynamic and end-organ response, together with device-related complications, should be repeatedly reassessed to guide recovery and de-escalation, escalation or transition to advanced heart replacement therapies. IABP, intra-aortic balloon pump; LV, left ventricular; LVAD, left ventricular assist device; mAFP, microaxial flow pump; MCS, mechanical circulatory support; RV, right ventricular; RVAD, right ventricular assist device; SCAI, Society for Cardiovascular Angiography and Interventions; VA-ECMO, veno-arterial extracorporeal membrane oxygenation.

Contemporary phenotyping should integrate aetiology, pathophysiology, severity and clinical profile (3). Relevant aetiological distinctions include AMI-CS, HF-CS, postcardiotomy CS and non-myocardial causes of CS, including valvular, pericardial, pulmonary vascular and arrhythmic causes (1). HF-CS may be further classified as *de novo* or acute-on-chronic, while ventricular involvement may be LV-, RV- or biventricular-predominant. Phenotype is dynamic and should be refined as additional echocardiographic, biochemical and invasive haemodynamic data become available (3). At the bedside, phenotyping should integrate echocardiography, systemic perfusion markers and, when available, invasive haemodynamics. LV-predominant shock is generally characterised by impaired LV function, reduced cardiac output or cardiac power and elevated PCWP; RV-predominant shock by RV dysfunction, elevated RAP/CVP relative to PCWP and reduced pulmonary artery pulsatility index. Biventricular shock combines both profiles. Lactate trajectory, vasoactive requirements, SVR, congestion and oxygenation should be interpreted serially rather than as isolated MCS implantation thresholds (3, 10).

Severity should be assessed in parallel with phenotype. The updated 2022 SCAI SHOCK classification provides a five-stage framework from stage A (at risk) to stage E (extremis), incorporating hypotension, hypoperfusion, biochemical abnormalities and treatment intensity (11). Serial reassessment is essential because clinical trajectory may be more informative than a single presentation stage.

Nevertheless, greater haemodynamic severity does not necessarily imply greater potential benefit from MCS. Advanced shock identifies patients with the greatest need for circulatory support, but also those in whom irreversible neurological injury, multiorgan failure, severe frailty or advanced comorbidity make recovery unlikely despite restoration of systemic flow (1, 3, 4, 7). Conversely, isolated thresholds for age, lactate concentration or shock stage should not be regarded as absolute determinants of candidacy.

CS is therefore most rational when circulatory support is expected to contribute to a credible therapeutic destination, such as myocardial recovery, surgical or percutaneous correction of a reversible structural or ischaemic cause, durable LVAD implantation or heart transplantation. When the definitive destination remains uncertain, MCS may instead serve as a bridge-to-decision strategy (3, 4, 7). The probability of myocardial recovery, reversibility of end-organ dysfunction and eligibility for advanced heart replacement therapies should be considered early rather than after prolonged support. Neurological status after cardiac arrest, however, requires particular caution because reliable prognostication may not be possible during the early phase. In the absence of meaningful recovery potential or a plausible definitive treatment strategy, MCS is unlikely to improve long-term outcome.

Patients resuscitated after cardiac arrest are particularly challenging. Myocardial stunning, vasoplegia and lactate accumulation from preceding no-flow or low-flow intervals may exaggerate circulatory severity, whereas hypoxic-ischaemic brain injury may determine prognosis independently of haemodynamic recovery (1, 12). Sedation, temperature management and metabolic disturbances may confound early neurological assessment. MCS candidacy should therefore distinguish established irreversible neurological injury from uncertain early neurological status and, when appropriate, allow time for appropriately timed multimodal neurological prognostication (13).

## 3 Contemporary evidence for MCS

Randomised controlled trials for MCS remain largely confined to acute myocardial infarction-related cardiogenic shock (AMI-CS). In IABP-SHOCK II, 600 patients with AMI-CS undergoing early revascularisation were randomised to intra-aortic balloon pump (IABP) support or standard care. Routine IABP use did not reduce 30-day mortality [39.7% vs. 41.3%; relative risk 0.96, 95% confidence interval (CI) 0.79–1.17], providing no evidence for routine use in AMI-CS (14).

Randomised evidence for routine VA-ECMO in CS has been neutral across EURO SHOCK (*n* = 35), ECMO-CS (*n* = 117) and ECLS-SHOCK (*n* = 420) (15–17). In the largest trial, ECLS-SHOCK, early VA-ECMO did not reduce 30-day mortality (47.8% vs. 49.0%; relative risk 0.98, 95% CI 0.80–1.19) and increased moderate or severe bleeding (23.4% vs. 9.6%) and peripheral vascular complications requiring intervention (11.0% vs. 3.8%) (17). An individual-patient-data analysis of DanGer-Shock-like patients similarly found no significant 6-month mortality benefit with VA-ECMO (18).

In contrast, DanGer-Shock provided the first randomised evidence of a survival benefit with active MCS in selected AMI-CS. Between 2013 and 2023, 360 patients with STEMI-CS at 14 centres in Denmark, Germany and the United Kingdom were randomised to Impella CP mAFP or standard care; 355 were analysed. The Impella CP provides up to approximately 3.5–3.6 L/min in clinical use (4). At 180 days, all-cause mortality was 45.8% with mAFP support and 58.5% with standard care (hazard ratio 0.74, 95% CI 0.55–0.99), although with more device-related adverse events and longer intensive care and hospital stay (19).

DanGer-Shock enrolled a highly selected STEMI-CS population. Preceding cardiac arrest occurred in approximately 20% of patients versus 78% in ECLS-SHOCK, but the trials also differed in eligibility criteria, shock phenotype, competing neurological risk, timing of randomisation and support, and trial setting. Their contrasting results should therefore not be interpreted as a direct comparison of mAFP and VA-ECMO efficacy (17, 19).

An individual-patient-data meta-analysis of nine randomised trials (*n* = 1,059) found no significant reduction in overall 6-month mortality with MCS. In a prespecified selection analysis of STEMI-CS with no or brief (<10 min) prior resuscitation, used as a proxy for reduced hypoxic brain injury risk, MCS was associated with lower 6-month mortality (hazard ratio 0.77, 95% CI 0.61–0.97). However, the analysis did not establish treatment-effect heterogeneity according to a prospectively defined shock phenotype and should be considered hypothesis-generating rather than validation of a phenotype-guided MCS strategy (8).

Evidence in HF-CS remains substantially weaker. Altshock-2, a multicentre randomised controlled trial, enrolled 101 patients with SCAI stage B–D HF-CS considered suitable for heart replacement therapy and compared early IABP implantation with standard care. The trial was stopped for futility at the prespecified interim analysis, with no significant improvement in 60-day survival or successful bridging to heart replacement therapy (81% vs. 75%; hazard ratio 0.72, 95% CI 0.31–1.68) (9). Whether other devices or selected HF-CS phenotypes may derive benefit remains uncertain.

Timing may also influence the clinical effect of MCS. Earlier restoration of cardiac output may reduce catecholamine requirements, improve oxygen delivery and limit progression to multiorgan dysfunction (3, 4), although no major randomised trial has specifically compared early versus delayed implantation. An observational meta-analysis of 13 studies including 6,810 patients with AMI-CS reported lower short-term mortality when Impella support was initiated before rather than during or after PCI (37.2% vs. 53.6%; relative risk 0.70, 95% CI 0.56–0.88). However, the observational nature of the included studies leaves these findings susceptible to selection bias and residual confounding (20).

Generating high-quality randomised evidence in CS is inherently challenging because of the time-critical nature and severity of the condition, difficulties surrounding recruitment and randomisation, and marked heterogeneity in aetiology, phenotype and clinical trajectory. DanGer-Shock itself required approximately 10 years of recruitment and expansion from Denmark to Germany and the United Kingdom to achieve target enrolment (19).

Taken together, contemporary evidence supports selective rather than routine use of MCS. Matching device physiology to shock phenotype is physiologically compelling, but remains a strategy informed by haemodynamic principles, subgroup observations and indirect evidence rather than one prospectively validated in randomised trials.

## 4 Types of MCS devices and phenotype-guided selection

MCS devices differ substantially in mechanism of action, vascular access, degree and duration of circulatory support, effects on ventricular loading and ability to provide respiratory support. Device selection should therefore reflect the predominant pathophysiological disturbance and anticipated clinical trajectory rather than achievable flow alone (4). Their main characteristics are summarised in Table 1.

**Table 1:** Main characteristics of short-term mechanical circulatory support devices in cardiogenic shock.

MCS device	Technique/usual access	Main haemodynamic effect	Respiratory support	Clinical scenario in which it may be considered	Main limitations and complications
IABP	Intra-aortic balloon counterpulsation; usually femoral arterial access. Axillary access may facilitate prolonged support and mobilisation.	Modest circulatory support; diastolic augmentation of coronary perfusion and reduction in LV afterload; dependent on native cardiac function and pulsatility.	No	Selected LV-predominant CS when modest support may be sufficient; bridge to definitive repair in selected post-AMI mechanical complications, particularly VSR or acute severe MR; LV unloading.	Limited haemodynamic support in severe CS; vascular injury, bleeding and limb ischaemia.
LV Mafp (e.g., Impella CP/5.5)	Transvalvular axial-flow pump from the LV to the ascending aorta. Impella CP: percutaneous large-bore arterial access; Impella 5.5: surgical graft, usually axillary.	Active LV support and unloading; increases systemic flow and reduces LV filling pressure, wall stress and myocardial oxygen demand. Impella CP: flow typically up to 3.6 L/min; short-term support (days). Impella 5.5: flow up to 5.5 L/min; suitable for longer support (weeks).	No	LV-predominant CS with adequate RV function and oxygenation when substantial circulatory support and LV unloading are required.	Requires adequate RV output to maintain LV preload; bleeding, vascular injury, limb ischaemia, haemolysis, device malposition and thrombosis. LV thrombus and a mechanical aortic valve preclude transvalvular LV mAFP support.
RV support (e.g., Impella RP or centrifugal-flow RVAD with ProtekDuo)	Impella RP: percutaneous axial-flow RV support, usually via femoral venous access. ProtekDuo: dual-lumen cannula, usually via the right internal jugular vein, connected to an extracorporeal centrifugal pump.	Active RV bypass/ unloading with increased pulmonary blood flow and LV preload.	Impella RP: no ProtekDuo-based RVAD: possible when an oxygenator is incorporated into the extracorporeal circuit	Predominant RV failure refractory to medical therapy when isolated LV support would be inadequate.	Device- and configuration-dependent; bleeding, vascular complications, haemolysis, thrombosis and cannula/device malposition; requires appropriate anatomy and expertise.
Peripheral VA-ECMO	Venous drainage and arterial return through an extracorporeal centrifugal pump and membrane oxygenator; peripheral femoral cannulation is most common.	High-flow circulatory support with right-heart unloading and systemic perfusion; retrograde arterial flow may increase LV afterload.	Yes	Refractory or profound circulatory failure requiring high-flow support, particularly when accompanied by biventricular dysfunction and/or severe respiratory failure, after considering trajectory, reversibility and therapeutic destination.	Bleeding, vascular injury and limb ischaemia, stroke, thrombosis, infection and haemolysis; increased LV afterload may cause LV distension and pulmonary oedema; differential hypoxaemia may occur with peripheral support.

CS, cardiogenic shock; IABP, intra-aortic balloon pump; LV, left ventricular; VSR, ventricular septal rupture; MR, mitral regurgitation; mAFP, microaxial flow pump; MCS, mechanical circulatory support; RV, right ventricular; RVAD, right ventricular assist device; VA-ECMO, veno-arterial extracorporeal membrane oxygenation. Device selection should integrate shock phenotype and trajectory, anticipated degree and duration of support, oxygenation requirements, vascular and anatomical feasibility, local expertise, recovery potential and therapeutic destination. Technical characteristics are summarised from references 4 and 21 of the manuscript.

The IABP augments diastolic coronary perfusion and reduces LV afterload, but provides only modest circulatory support dependent on native cardiac function and pulsatility. Femoral access is most common, whereas axillary placement may facilitate prolonged support and mobilisation (4). Accordingly, IABP may be considered in selected LV-predominant CS when modest support is sufficient or as a bridge to definitive treatment in post-AMI mechanical complications, particularly ventricular septal rupture or acute severe mitral regurgitation.

The mAFPs provide active LV support by aspirating blood directly from the LV and expelling it into the ascending aorta. By increasing systemic flow while unloading the LV, they reduce LV end-diastolic pressure, ventricular wall stress and myocardial oxygen demand (4). The Impella CP provides percutaneous short-term support through large-bore arterial access, with flow in clinical use typically up to approximately 3.5–3.6 L/min, whereas the Impella 5.5 can provide flow up to 5.5 L/min and is suitable for longer support, usually through a surgically implanted axillary graft (4). In LV-predominant CS with adequate RV function and oxygenation, mAFP support may therefore be considered when substantial circulatory support and LV unloading are required. However, adequate RV output is necessary to maintain LV preload, and isolated LV mAFP support does not provide oxygenation. LV thrombus and a mechanical aortic valve prosthesis preclude transvalvular LV mAFP support, while vascular anatomy and bleeding risk may further limit feasibility (4, 7).

Predominant RV failure requires a different support strategy because isolated LV mAFP support relies on adequate RV output. Dedicated RV support may be provided using an axial-flow device such as the Impella RP or a centrifugal-flow RV assist configuration with a dual-lumen ProtekDuo® cannula (21). These devices bypass or unload the failing RV and increase pulmonary blood flow and LV preload; an oxygenator may be incorporated into selected centrifugal circuits when additional respiratory support is required.

Veno-arterial extracorporeal membrane oxygenation (VA-ECMO) provides high-flow circulatory support and gas exchange and may therefore be considered in refractory or profound circulatory failure requiring high-flow support, particularly when accompanied by biventricular dysfunction or severe respiratory failure (4, 7). VA-ECMO can be established centrally or peripherally, although peripheral femoral venous and arterial cannulation is most common. Venous drainage unloads the right heart, whereas arterial return restores systemic perfusion. With peripheral VA-ECMO, however, retrograde aortic flow may increase LV afterload, resulting in LV distension, elevated filling pressures, pulmonary congestion and impaired aortic valve opening (3, 4). Selected patients may consequently require additional LV unloading.

LV unloading may be considered in the presence of progressive LV distension, persistent aortic valve closure, pulmonary oedema, elevated filling pressures or intracardiac/aortic-root stasis. Unloading may be instituted upfront or as a rescue strategy when these features develop. Available approaches include IABP, which provides modest indirect unloading; mAFP, which provides active LV unloading; and transseptal left-atrial drainage or surgical LV venting. Device choice should balance the required degree of unloading against procedural complexity and the additional bleeding and vascular risks of combined-device support. Importantly, evidence supporting routine LV unloading during VA-ECMO remains limited, and a universal survival benefit has not been established (4, 21, 22).

Large-bore arterial cannulation also necessitates careful assessment of distal limb perfusion, and placement of a distal perfusion cannula should be considered to reduce limb ischaemia.

Device choice is particularly complex in mixed or evolving shock phenotypes. In mixed cardiogenic–vasodilatory shock, increasing mechanical flow alone may not correct hypoperfusion when reduced systemic vascular resistance remains important (10). Technical feasibility may further limit the physiologically preferred device: peripheral arterial disease, aortic pathology, LV thrombus, mechanical aortic valve prostheses, coagulopathy or limited vascular access may preclude specific strategies (4, 7). Final selection should integrate ventricular and respiratory phenotype, required duration of support, anatomical feasibility, local expertise and therapeutic destination.

## 5 Complications in the intensive care unit

Contemporary randomised controlled trials illustrate the complication burden of MCS. In DanGer-Shock, moderate or severe bleeding occurred in 21.8% of patients receiving mAFP support compared with 11.9% of controls, while limb ischaemia occurred in 5.6% and 1.1%, respectively (19).

Haemostatic disturbances during MCS are multifactorial and may promote both bleeding and thrombosis. Contact between blood and non-endothelialised artificial surfaces activates the contact coagulation pathway, platelets and complement, promoting thrombin generation and inflammation (23). Conversely, prolonged device exposure may result in consumption of platelets and coagulation factors (4). High shear stress can additionally impair platelet function and induce an acquired von Willebrand syndrome through loss of high-molecular-weight von Willebrand factor multimers (24). In VA-ECMO, exposure to the oxygenator may promote fibrinolysis, further contributing to the haemorrhagic phenotype (23).

These device-related mechanisms interact with systemic inflammation, therapeutic anticoagulation and critical-illness-related organ dysfunction, including impaired hepatic synthesis of coagulation factors (21, 23). Consequently, anticoagulation must be individualised and repeatedly reassessed according to both thrombotic and haemorrhagic risk (4, 21, 23).

Thrombotic and ischaemic complications result from a similarly complex interaction between patient- and device-related factors. Artificial surfaces and abnormal flow patterns promote coagulation, whereas inadequate flow or blood stasis may favour intracardiac or circuit thrombosis. Large-bore arterial access can cause limb ischaemia and vascular injury, requiring continuous assessment of distal limb perfusion; antegrade distal perfusion cannula placement should be considered at peripheral VA-ECMO implantation (4, 21). Thromboembolic events may also involve the cerebral or mesenteric circulation. During VA-ECMO, inadequate LV ejection may result in progressive LV distension and intracavitary blood stasis, increasing the risk of LV or aortic-root thrombosis in addition to pulmonary congestion. Selected patients with LV distension, pulmonary congestion or blood stasis may consequently require LV unloading (4, 21).

Other complications are device specific. Haemolysis is particularly relevant with high-speed mAFPs because of shear stress and may be aggravated by malposition or suction events (4). Surveillance should include device position and function together with haemoglobin, lactate dehydrogenase, plasma-free haemoglobin or haptoglobin when haemolysis is suspected. Bedside echocardiography is particularly useful for identifying mAFP malposition and assessing LV distension, thrombus formation and aortic valve opening during VA-ECMO (21). Peripheral VA-ECMO may additionally cause differential hypoxaemia, also known as Harlequin syndrome, when poorly oxygenated native LV output supplies the upper body whereas oxygenated extracorporeal flow preferentially perfuses the lower body. Pump thrombosis, oxygenator dysfunction and mechanical injury related to cannula or device malposition should also be actively considered.

Infectious complications become increasingly relevant with prolonged intravascular support and are influenced by support duration and vascular access site rather than by the pump alone (21). In DanGer-Shock, sepsis with a positive blood culture occurred in 11.7% of patients receiving mAFP support compared with 4.5% of controls (19). Strict aseptic management of cannulation and vascular access sites and a low threshold for investigation of suspected infection are therefore important, although routine prophylactic antibiotics are not recommended for most MCS configurations (21).

Complication prevention and surveillance should begin before implantation with assessment of vascular anatomy, haemostatic status, anticoagulation requirements and anticipated duration of support. Continued exposure should be justified by haemodynamic benefit or a defined bridge strategy, with daily reassessment of myocardial recovery, end-organ perfusion, device-related complications and readiness for de-escalation. Concomitant use of more than one MCS device may provide additional haemodynamic benefit but further increases bleeding and vascular risk (4). When adequate recovery has been achieved, support should be reduced in a device-specific, stepwise manner and explantation pursued as soon as clinically appropriate (21).

## 6 Evidence gaps and future directions

Important evidence gaps remain regarding MCS in non-AMI and HF-related CS, optimal timing of implantation, phenotype-specific device selection and strategies for escalation and de-escalation. High-quality evidence remains dominated by AMI-CS, whereas the role of MCS across the heterogeneous spectrum of HF-CS and other non-ischaemic aetiologies is substantially less well defined (1, 4). Future studies should therefore avoid considering CS as a single therapeutic entity and instead identify populations in whom the physiological effect of a specific device is most likely to translate into meaningful clinical benefit.

Future clinical trials should move beyond traditional device-versus-control comparisons towards phenotype- and strategy-based designs accounting for aetiology, SCAI stage and trajectory, ventricular and respiratory phenotype, cardiac-arrest status, neurological prognosis, recovery potential, vascular feasibility and therapeutic destination, with prespecified operational haemodynamic and echocardiographic criteria rather than broad clinical labels.

Relevant questions include when support should be initiated, when escalation or combined-device support is justified, how and when support should be de-escalated, and whether improved device design and complication-prevention strategies can reduce bleeding, vascular injury, thrombosis, haemolysis and infection. Outcomes should extend beyond short-term mortality to include successful bridging to recovery or advanced heart replacement therapies, neurological and functional recovery, and resource use.

Progress will also depend on systems of care capable of delivering complex decisions rapidly. Multidisciplinary shock teams and regionalised networks may facilitate early phenotyping, referral to experienced centres, appropriate device selection, complication management and transition to advanced heart failure therapies, although the evidence supporting these organisational strategies remains predominantly observational (1, 21, 25). MCS should therefore be regarded not as an isolated procedure but as one component of a coordinated and repeatedly reassessed intensive care strategy. The future of the field lies less in providing more support to more patients than in identifying those in whom the right support, delivered with the right technique, at the right moment and with a realistic therapeutic strategy, can meaningfully alter the course of shock.

## References

[1] Thiele H Hassager C. Cardiogenic shock. N Engl J Med. (2026) 394(1):62–77. 10.1056/NEJMra2312086 41467651

[2] Sinha SS Morrow DA Kapur NK Kataria R Roswell RO. 2025 Concise clinical guidance: an ACC expert consensus statement on the evaluation and management of cardiogenic shock. J Am Coll Cardiol. (2025) 85(16):1618–41. 10.1016/j.jacc.2025.02.018 40100174

[3] Lim HS González-Costello J Belohlavek J Zweck E Blumer V Schrage B. Hemodynamic management of cardiogenic shock in the intensive care unit. J Heart Lung Transplant. (2024) 43(7):1059–73. 10.1016/j.healun.2024.03.009 38518863 PMC11148863

[4] Møller JE Thiele H Morrow D Kjærgaard J Hassager C. Mechanical circulatory support: when, how, and for whom. Eur Heart J. (2025) 46(16):1480–92. 10.1093/eurheartj/ehae925 39791535

[5] Berg DD Bohula EA Patel SM Alfonso CE Alviar CL Baird-Zars VM. Epidemiology of cardiogenic shock using the shock academic research consortium (SHARC) consensus definitions. Eur Heart J Acute Cardiovasc Care. (2024) 13(10):709–14. 10.1093/ehjacc/zuae098 39208447 PMC11518926

[6] García-García C López-Sobrino T Sanz-Girgas E Cueto MR Aboal J Pastor P. Cardiogenic shock mortality according to aetiology in a Mediterranean cohort: results from the shock-CAT study. ESC Heart Fail. (2025) 12(2):1336–45. 10.1002/ehf2.15148 PMC11911611 39587711

[7] Grant MC Brudney CS Hernandez-Montfort J Ibekwe SO Rea A Stoppe C. Definitions of cardiogenic shock and indications for temporary mechanical circulatory support: joint consensus report of the PeriOperative quality initiative and the enhanced recovery after surgery cardiac society. Ann Thorac Surg. (2025) 120(2):202–12. 10.1016/j.athoracsur.2025.01.040 40187589

[8] Thiele H Møller JE Henriques JPS Bogerd M Seyfarth M Burkhoff D. Temporary mechanical circulatory support in infarct-related cardiogenic shock: an individual patient data meta-analysis of randomised trials with 6-month follow-up. Lancet. (2024) 404(10457):1019–28. 10.1016/S0140-6736(24)01448-X 39236726

[9] Morici N Sacco A Frea S Rota M Villanova L Gravinese C. Early intra-aortic balloon support for heart failure-related cardiogenic shock: a randomized clinical trial. J Am Coll Cardiol. (2025) 85:1587–97. 10.1016/j.jacc.2025.03.003 40162941

[10] Van Diepen S Pöss J Senaratne JM Gage A Morrow DA. Mixed cardiogenic shock: a proposal for standardized classification, a hemodynamic definition, and framework for management. Circulation. (2024) 150:1459–68. 10.1161/CIRCULATIONAHA.124.069508 39466889

[11] Naidu SS Baran DA Jentzer JC Hollenberg SM Van Diepen S Basir MB. SCAI SHOCK stage classification expert consensus update: a review and incorporation of validation studies. J Am Coll Cardiol. (2022) 79:933–46. 10.1016/j.jacc.2022.01.018 35115207

[12] Izquierdo Ribas M López-Sobrino T Moreno E De Diego O Roca C Cepas P. Predictive value of bicarbonate levels at admission for severe hypoxic-ischemic encephalopathy after out-of-hospital cardiac arrest. Med Clin (Barc). (2025) 165(2):106977. 10.1016/j.medcli.2025.106977 40412100

[13] Nolan JP Sandroni C Cariou A Cronberg T D’Arrigo S Haywood K. European resuscitation council and European society of intensive care medicine guidelines 2025 post-resuscitation care. Resuscitation. (2025) 215(Suppl 1)):110809. 10.1016/j.resuscitation.2025.110809 41117575

[14] Thiele H Zeymer U Neumann F-J Ferenc M Olbrich H-G Hausleiter J. Intraaortic balloon support for myocardial infarction with cardiogenic shock. N Engl J Med. (2012) 367:1287–96. 10.1056/NEJMoa1208410 22920912

[15] Banning AS Sabaté M Orban M Gracey J López-Sobrino T Massberg S. Venoarterial extracorporeal membrane oxygenation or standard care in patients with cardiogenic shock complicating acute myocardial infarction: the multicentre, randomised EURO SHOCK trial. Euro Intervention. (2023) 19(6):482–92. 10.4244/EIJ-D-23-00204 37334659 PMC10436068

[16] Ostadal P Rokyta R Karasek J Kruger A Vondrakova D Janotka M. Extracorporeal membrane oxygenation in the therapy of cardiogenic shock: results of the ECMO-CS randomized clinical trial. Circulation. (2023) 147(6):454–64. 10.1161/CIRCULATIONAHA.122.062949 36335478

[17] Thiele H Zeymer U Akin I Behnes M Rassaf T Mahabadi AA. Extracorporeal life support in infarct-related cardiogenic shock. N Engl J Med. (2023) 389:1286–97. 10.1056/NEJMoa2307227 37634145

[18] Zeymer U Freund A Hochadel M Ostadal P Belohlavek J Massberg S. Do DanGer-SHOCK-like patients benefit from VA-ECMO treatment in infarct-related cardiogenic shock? Results of an individual patient data meta-analysis. Eur Heart J Acute Cardiovasc Care. (2024) 13(9):658–61. 10.1093/ehjacc/zuae093 39217624

[19] Møller JE Engstrøm T Jensen LO Eiskjær H Mangner N Polzin A. Microaxial flow pump or standard care in infarct-related cardiogenic shock. N Engl J Med. (2024) 390:1382–93. 10.1056/NEJMoa2312572 38587239

[20] Iannaccone M Franchin L Hanson ID Boccuzzi G Basir MB Truesdell AG. Timing of impella placement in PCI for acute myocardial infarction complicated by cardiogenic shock: an updated meta-analysis. Int J Cardiol. (2022) 362:47–54. 10.1016/j.ijcard.2022.05.011 35533755

[21] Geller BJ Sinha SS Kapur NK Bakitas M Balsam LB Chikwe J. Escalating and de-escalating temporary mechanical circulatory support in cardiogenic shock: a scientific statement from the American Heart Association. Circulation. (2022) 146:e50–68. 10.1161/CIR.0000000000001076 35862152

[22] Kim MC Lim Y Lee SH Shin Y Ahn JH Hyun DY. Early left ventricular unloading or conventional approach after venoarterial extracorporeal membrane oxygenation: the EARLY-UNLOAD randomized clinical trial. Circulation. (2023) 148(20):1570–81. 10.1161/CIRCULATIONAHA.123.066179 37850383

[23] Zeibi Shirejini S Carberry J McQuilten ZK Burrell AJC Gregory SD Hagemeyer CE. Current and future strategies to monitor and manage coagulation in ECMO patients. Thromb J. (2023) 21(1):11. 10.1186/s12959-023-00452-z 36703184 PMC9878987

[24] Frere C Mazzeffi M Maier CL Helms J Steiner ME Sullenger BA. Acquired von willebrand syndrome during extracorporeal membrane oxygenation support: a comprehensive review of current evidence: communication from the ISTH SSC on perioperative and critical care thrombosis and hemostasis. J Thromb Haemost. (2024) 22(9):2608–28. 10.1016/j.jtha.2024.06.007 38925492

[25] Papolos AI Kenigsberg BB Berg DD Alviar CL Bohula E Burke JA. Management and outcomes of cardiogenic shock in cardiac ICUs with versus without shock teams. J Am Coll Cardiol. (2021) 78:1309–17. 10.1016/j.jacc.2021.07.044 34556316

